# Perfluorononanoic Acid (PFNA) Exacerbates Atopic Dermatitis by Inducing Inflammation in Mice

**DOI:** 10.3390/toxics13070585

**Published:** 2025-07-13

**Authors:** Jiali Xiao, Junchao Wang, Nuo Xu, Xulong Huang, Farid Khalilov, Xianfeng Huang, Xiangyong Zheng, Xiashun Xu, Shisheng Lin, Wengang Zhao, Elchin Khalilov

**Affiliations:** 1 College of Life and Environmental Sciences, Wenzhou University, Wenzhou 325000, China; niaoniao200008@163.com (J.X.); 20160125@wzu.edu.cn (N.X.); 13780196760@163.com (X.H.); farid.khalilov.87@mail.ru (F.K.); xianfeng_huang@wzu.edu.cn (X.H.); x.zheng@wzu.edu.cn (X.Z.); 15067853575@163.com (X.X.); 19874499115@163.com (S.L.); 2 School of Pharmaceutical Sciences, Wenzhou Medical University, Wenzhou 325000, China; junchaowang2024@163.com

**Keywords:** Perfluorononanoic acid, atopic dermatitis, inflammatory, MAPK

## Abstract

Perfluorononanoic acid (PFNA) is a ubiquitous persistent environmental pollutant, and several studies have found significant links between atopic dermatitis (AD) and prenatal exposure to PFNA. However, the relationship between PFNA and AD remains unclear. In this study, 2,4-dinitrochlorobenzene (DNCB)-treated female BALB/c mice were used as AD models to investigate the effects of PFNA and its potential mechanisms. These mice were topically applied with 5 mg/kg PFNA per day for 15 days. The results demonstrated that PFNA significantly increased AD lesion severity and clinical symptoms, including dermatitis score, ear thickness, and epidermal thickness. In addition, PFNA also increased the serum IgE level, splenic atrophy, and upregulated the expression of *TNF*-*α*, *IL*-*6*, and *IL*-*1β*, genes that are associated with skin inflammatory factors. In addition, Western blot results showed that PFNA treatment upregulated the expression of p-JNK protein. Additionally, cellular experiments indicated that RAW264.7 macrophages and mouse brain microvascular endothelial (bEnd.3) cells treated with PFNA at concentrations of 0.01–100 μM for 72 h showed no changes in cell viability. However, 100 μM PFNA upregulated the mRNA expression levels of the pro-inflammatory cytokines IL-1β and IL-6, as well as the protein expression of p-JNK, in RAW264.7 cells induced with 1 mg/mL LPS for 2 h. Similarly, PFNA increased TNF-α and IL-6 mRNA expression and p-JNK protein expression in bEnd.3 cells stimulated with 20 ng/mL TNF-α for 0.5 h. Based on these findings, we can conclude that PFNA may aggravate atopic dermatitis by promoting inflammation.

## 1. Introduction

Perfluoroalkyl substances (PFASs) are extensively used in various applications, including food contact materials, textiles, firefighting foam, surfactants, insecticides, pesticides, and others, because of their water-, heat-, stain-, and grease-resistant properties [1,2]. However, the strong carbon–fluorine bonds in residual PFASs confer an unparalleled stability, rendering them resistant to natural degradation processes and enabling multi-decadal environmental persistence [3]. In recent years, the prevalence of PFASs in the environment has caused widespread concern, with increasing evidence that PFASs are toxic to both humans and animals, making them a major health issue of global concern [4]. Perfluorononanoic acid (PFNA), a representative of the PFAS family, is a 9-carbon compound that is widely distributed in the environment, and as it is difficult to degrade, it can persist for a long period in water, soil, and organisms. Recent studies have shown that PFNA does not only widely contaminate the environment, but is also toxic to human health and the ecosystem as a whole. The toxicity of PFNA includes reproductive toxicity [5], hepatotoxicity [6], developmental toxicity [7], and immunotoxicity [8].

The immune system serves as the body’s defense barrier against harmful substances from the external environment, and an abnormality or a disorder in its functioning may lead to the onset and development of a wide range of diseases [9]. Inflammation is one of the basic responses of the immune system to infection, injury, or exposure to toxins, and prolonged or excessive inflammation can lead to the development of chronic diseases. A chronic inflammatory skin condition, atopic dermatitis (AD) is common across the globe. Patients suffering from it typically experience intense itching and repeated occurrences of skin lesions [10]. In industrialized countries, the prevalence of AD has increased from two- to threefold, affecting from about 15 to 20% of children and from 1 to 3% of adults globally [11,12]. Its pathogenesis has not been fully elucidated, but the interplay of genetic susceptibility, immune system disorders, and environmental factors is thought to play a key role in its onset and exacerbation. Prenatal exposure to PFNA has been found to increase the probability of AD in children [13]. Most of the available studies have focused on the effect of PFNA on the immune system, but further research is still needed to better understand its role in AD disease. Given the widespread environmental contamination of PFNA and its potential impact on the immune system, exploring how PFNA worsens the inflammatory response and triggers chronic diseases is of great scientific and social importance.

This study investigated the effects of PFNA on inflammatory responses and aimed to provide a theoretical basis for further understanding the potential risks of PFNA and other PFAS analogs on the immune system and inflammation. The findings presented here may constitute a scientific basis for the development of environmental pollution control and public health policies.

## 2. Methods

### 2.1. Materials

1-Chloro-2,4-dinitrobenzene (DNCB), LPS, and PFNA (97% purity; M.W. 464 g/mol) were purchased from Sigma-Aldrich Co. TNF-α and INF-γ were purchased from Thermo Fisher Scientific (Waltham, MA, USA).

### 2.2. Animals

Seven-week-old female BALB/c mice (17–20 g body weight) were purchased from Gempharmatech Co., Ltd. (Nanjing, China), and the age of the mice was referred to Park et al. [14] and Lee et al. [15], in which the dorsal skin barrier was intact during this time period. The animals were kept at 22 ± 1 °C under 50–55% relative humidity and a 12 h light/dark cycle. All animal experiments (WZU-2024-111) adhered to international ethical standards and the guidelines set by the National Institutes of Health for laboratory animal care and use. Prior to commencement, all experimental protocols underwent review and received approval from the Wenzhou University Animal Care and Use Committee. Animal experiments were conducted according to the ARRIVE guidelines.

### 2.3. Induction of AD Using DNCB

After one week of acclimatization to the laboratory conditions stated above, the mice were randomly divided into three groups (n = 6, respectively). One group was designated as the healthy group, and it received no treatment. The other two groups were treated with DNCB to induce the condition of AD by injecting the dorsal skin with 100 μL of 7% DNCB for sensitization. After one day, the dorsal skin and the inside and outside of the right ear were each treated with 100 μL of 1% DNCB once a day for four days. Day 1 was considered the day of sensitization. Four days prior to sensitization, one of the DNCB groups was designated as the AD group without treatment, while the other AD group then received oral gavage with PFNA (Sigma-Aldrich, St., Louis, MO, USA) at a dose of 5 mg/kg/day over 15 days, and this group was designated as the AD + PFNA group. Two days prior to sensitization, the hairs on the back of the skin (approximately 2 cm × 3 cm in area) were removed by shaving with an electric shaver. Afterwards, all mice were euthanized with isoflurane and spleen, serum, and skin were collected.

### 2.4. The Measure of Basic Characteristics in BALB/c Mice

Before each sensitization, the skin and ears were photographed using a camera, and the thickness of the right and left ears of a pair of mice was measured using a vernier caliper. Concurrent with experimental observations, dermatological manifestations were systematically evaluated through a standardized clinical scoring protocol. This quantitative scaling system categorized cutaneous pathology into the following five distinct parameters: erythematous lesions/hemorrhagic foci, edematous swelling/hematoma formation, excoriation/erosion, pruritic intensity/xerosis severity, and dermal lichenification patterns. Asymptomatic was assigned as 0, a mild symptom as 1, a medium symptom as 2, and a severe symptom as 3 [16]. Additionally, the spleen was removed, weighed, and photographed using a camera.

### 2.5. Measure of Serum IgE Level in BALB/c Mice

Blood samples were collected, centrifuged, and the supernatant was preserved for subsequent quantitative assessment of serum IgE levels via an IgE ELISA kit (Xinbosheng, Shenzhen, China), strictly adhering to the manufacturer’s protocol.

### 2.6. Histological Analysis of BALB/c Mice

Immediate euthanasia of the mice was performed at the end of blood sampling. The obtained back skin and ears were fixed in 4% paraformaldehyde. The samples were embedded in paraffin, sectioned into 6 μm slices, and then stained with H&E and toluidine blue, followed by examination with a DM3000 microscope (Leica Microsystems GmbH, Wetzlar, Germany).

### 2.7. Immunofluorescence Staining Assay

After treatment with 3% H_2_O_2_ and blocking with 5% bovine serum albumin (BSA), paraffin-fixed tissue sections were incubated with PCNA antibody (Cell Signaling Technology, Beverly, MA, USA) at 4 °C overnight. Afterwards, the samples underwent incubation with Alexa Fluor 568 anti-rabbit secondary antibody (Thermo Fisher Scientific, Waltham, MA, USA). Subsequently, they were sealed using an anti-fluorescence quencher with DAPI (Thermo Fisher Scientific, Waltham, MA, USA). Fluorescence changes were then monitored using a confocal laser scanning microscope (Olympus, Tokyo, Japan).

### 2.8. Cell Culture

The RAW264.7 (murine macrophage) cell line and bEnd.3 (a mouse brain endothelial) cell line were obtained from the Cell Bank of the Chinese Academy of Sciences (Shanghai, China), and the human keratinocyte cell line, HaCaT, was purchased from Sciencell (San Diego, CA, USA). The cells were maintained in DMEM (GIBCO, Life Technologies Corporation, New York, NY, USA) supplemented with 10% fetal bovine serum (FBS) (GIBCO, Life Technologies Corporation, New York, NY, USA), 100 U/mL penicillin, and 100 U/mL streptomycin under standard incubation conditions (37 °C, 5% CO_2_, humidified atmosphere). The cells were treated with PFNA for 72 h.

### 2.9. Calcein-AM/PI Double Staining Assay

The RAW264.7 and bEnd.3 cell lines were exposed to PFNA at graded concentrations (0.01, 0.1, 1, 10, and 100 μM) over a 72 h experimental period, referencing the experimental protocol from Paula Pierozan et al. [17]. After incubation, the cells were resuspended in PBS and cultured in fresh DMEM at 37 °C for 0.5 h in a 5% CO_2_ incubator. Next, the cells were washed with PBS, digested with trypsin and then centrifuged at 1000× *g* for 3 min. Following PBS-based resuspension to 10^5^–10^6^ cells/mL, calcein-AM staining solution (Dojindo Laboratories, Tokyo, Japan) was administered at a 2.5 μM concentration with 37 °C incubation. Dual-channel fluorescence detection (Carl Zeiss AG, Oberkochen, Germany) enabled the discrimination of live (green) versus dead (red) cellular states, with subsequent ImageJ 1.54 software-mediated data analysis.

### 2.10. Real-Time Quantitative PCR

The total RNA from a skin sample was isolated with TRIzol Reagents (Thermo Fisher Scientific, Waltham, MA, USA) according to the manufacturer’s instructions. Total RNA was extracted from RAW264.7 macrophages and bEnd.3 cells using TRIZOL reagent (Vazyme Biotech Co., Nanjing, China) [18], and the sample concentration was measured using NanoDrop (Thermo Fisher Scientific, Waltham, MA, USA). The obtained RNA was reverse transcribed into cDNA using the Prime Script RT Enzyme Cutting Kit (Takara, Dalian, China) to reverse transcribe 1 μg of RNA into cDNA. The obtained cDNA was subjected to real-time quantitative PCR (RT-qPCR) using SYBR Green Mix (Applied Biosystems, Foster City, CA, USA). The expression levels of genes were standardized with the housekeeping gene GAPDH. The 2^−ΔΔCt^ method was employed to analyze the relative expression of each target gene. All the primers utilized in RT-qPCR are detailed in Table 1.

### 2.11. Western Blot

Skin extract preparation was initiated by mincing tissue samples into fragments, which were subsequently subjected to mechanical homogenization in RIPA Lysis Buffer (Beyotime, Shanghai, China) supplemented with a 1% (*v*/*v*) protease–phosphatase inhibitor cocktail. As for RAW264.7 macrophages and bEnd.3 cells, the cells were lysed in Glo Lysis Buffer (Promega, Madison, WI, USA) containing protease inhibitors and phosphatase inhibitors. Both the skin and cell extracts were centrifuged, and the supernatants, which contained the soluble proteins, were subjected to a protein assay using a commercial BCA kit (Yisheng, Shanghai, China). Equal protein quantities were resolved via SDS-PAGE. Protein bands from the gel were then electrotransferred to a PVDF membrane (Millipore, Billerica, MA, USA). The membrane was first blocked with 5% no-fat milk in TBST buffer (10 mM Tris-HCl (pH 6.8), 100 mM NaCl, and 1% Tween 20). Next, it was washed and then incubated with primary antibody, as described in Table 2, overnight at 4 °C. After that, the blot was washed and incubated with the corresponding secondary antibody for 4 h at room temperature. Finally, the blot was washed again and then subjected to chemiluminescence assay (Pierce, Rockford, IL, USA), and images of the blot were acquired using an Amersham Imager (GE Healthcare Biosciences, Pittsburgh, PA, USA).

### 2.12. Statistical Analysis

Statistical analyses were conducted with GraphPad Prism 9.0 software (GraphPad, San Diego, CA, USA). Data are indicated as the mean ± standard deviation of three replicates. Statistical differences between groups were assessed using analysis of variance (ANOVA), and correction for multiple comparisons was made using Dunnett’s test. In all comparisons, *p*-values less than 0.05 were considered statistically significant. Significance and n values are provided in the figure legends.

## 3. Results

### 3.1. Effects of PFNA on DNCB-Induced Skin Lesions in BALB/c Mice

**Figure 1:**
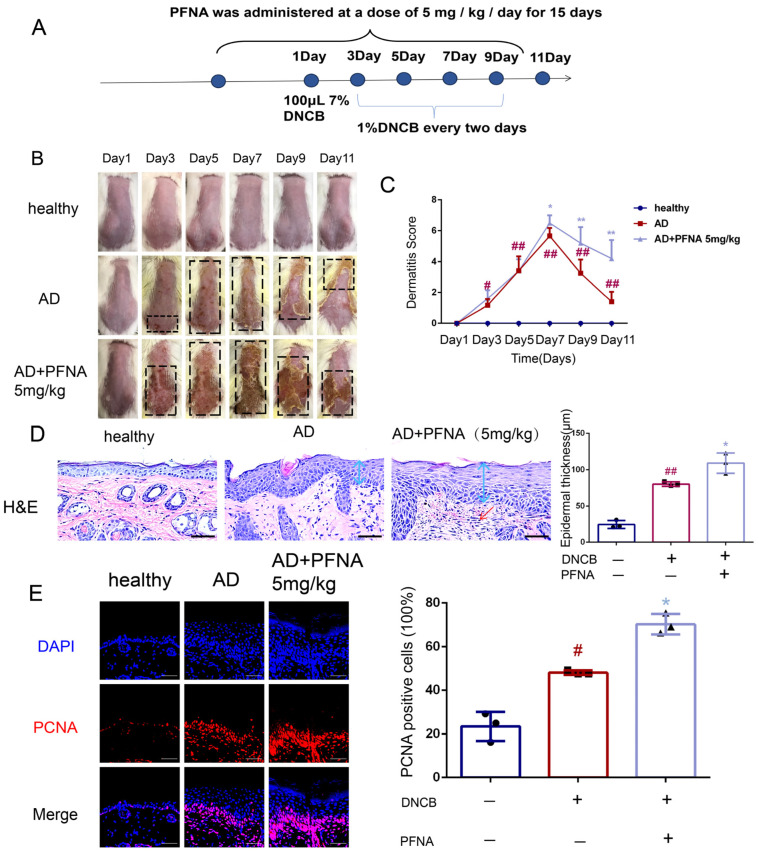
Role of PFNA in DNCB-induced atopic dermatitis in vivo. Representative photographs and histopathological images of the dorsal skin lesions on the backs of DNCB-induced AD mice. (**A**) Experimental schedule for the induction of AD-like skin lesions. (**B**) Photographs taken of the back skin showing AD-like skin damage. (**C**) Dermatitis severity score calculated as the aggregate of individual scores (n = 4–6). (**D**) Sections of AD dorsal skin lesions with HE staining (magnification ×20, scale bar: 100 µm) and determination of epidermal thickness (n = 3). Blue arrows indicate epidermal thickness, while red arrows denote inflammatory cell infiltration (**E**) Immunofluorescence labeling images of PCNA, a skin proliferation marker, on the backs of mice. The plot beside the image shows a quantitative analysis of PCNA-positive cells among the different groups (n = 3). Data in the graphs are shown as the mean ± SD (n = 3). ^#^ *p* < 0.05 and ^##^ *p* < 0.01 vs. the healthy group, * *p* < 0.05 and ** *p* < 0.01 vs. the AD group.

An AD mouse model was developed in BALB/c mice through DNCB induction to assess PFNA’s effects, with the experimental design outlined in Figure 1A. Typical AD symptoms such as erythema and scabbing were observed on the skin of the mice in the AD group from day 5 onwards, with a significant increase in erythema and scabbing, which continued until the end of day 11 (Figure 1B). Histopathological examination showed that the AD + PFNA group exhibited a significant increase in the thickness of the skin (Figure 1C). The thickening of the epidermis and dermis found in the AD group of mice was significantly increased compared with the mice in the healthy group, and this thickening was significantly increased after PFNA treatment (Figure 1C). In addition, the clinical skin score of the AD group increased significantly after induction, and the severity of dermatitis in the AD + PFNA group increased significantly after seven days (Figure 1D). Immunofluorescence analysis of the skin revealed significantly more PCNA-positive cells in the AD group than in the healthy group, and more PCNA-positive cells appeared in the AD + PFNA group (Figure 1E). Overall, this result provides support that PFNA can worsen AD-like symptoms in mice.

### 3.2. PFNA Aggravated AD-Like Lesions in the Right Ears of Mice

**Figure 2:**
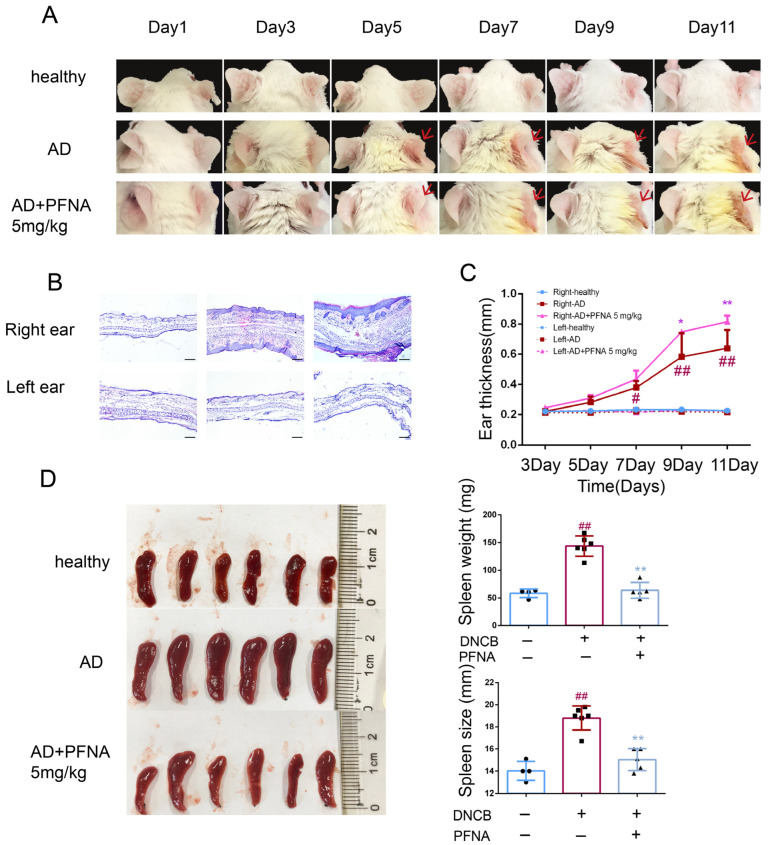
Effect of PFNA on ear thickness and spleen hypertrophy. (**A**) Photographs of ear skin lesions in DNCB-induced AD mice. (**B**) Sections of AD ear skin lesions, HE-stained (magnification ×10, scale bar: 100 µm) (n = 3). (**C**) Ear skin thickness measurements were taken with digital calipers (n = 4–6). (**D**) Photographs of spleen, spleen weight, and spleen length in DNCB-induced AD mice. Data in the plots are shown as the mean ± SD (n = 4–6). ^#^ *p* < 0.05 and ^##^ *p* < 0.01 vs. the healthy group, * *p* < 0.05 and ** *p* < 0.01 vs. the AD group.

Typical symptoms of AD were observed in the right ears of the mice in the AD group, such as a significant increase in thickness and erythema (Figure 2A). Mice in the AD + PFNA group displayed a significantly increased thickness of the right ear throughout the experimental period (Figure 2C). Histopathological examinations showed that PFNA significantly worsened symptoms and increased the right ear thickness of the mice in the AD group (Figure 2B).

### 3.3. Effects of PFNA on DCNB-Induced Spleen in Mice

In order to determine whether PFNA affected the systemic immune response of the AD mice, we next examined the spleen to quantify the atopic symptoms in the mouse model [19]. AD usually produces a systemic immune response by affecting the immune organs [20]. The weight and volume of the lymph nodes and spleen of the mice in the AD group were greater compared with those in the healthy group. However, the mice in the AD + PFNA group showed a decrease in the weight and size of the spleen (Figure 2D).

### 3.4. PFNA Aggravates DNCB-Induced Inflammation in BALB/c Mice

**Figure 3:**
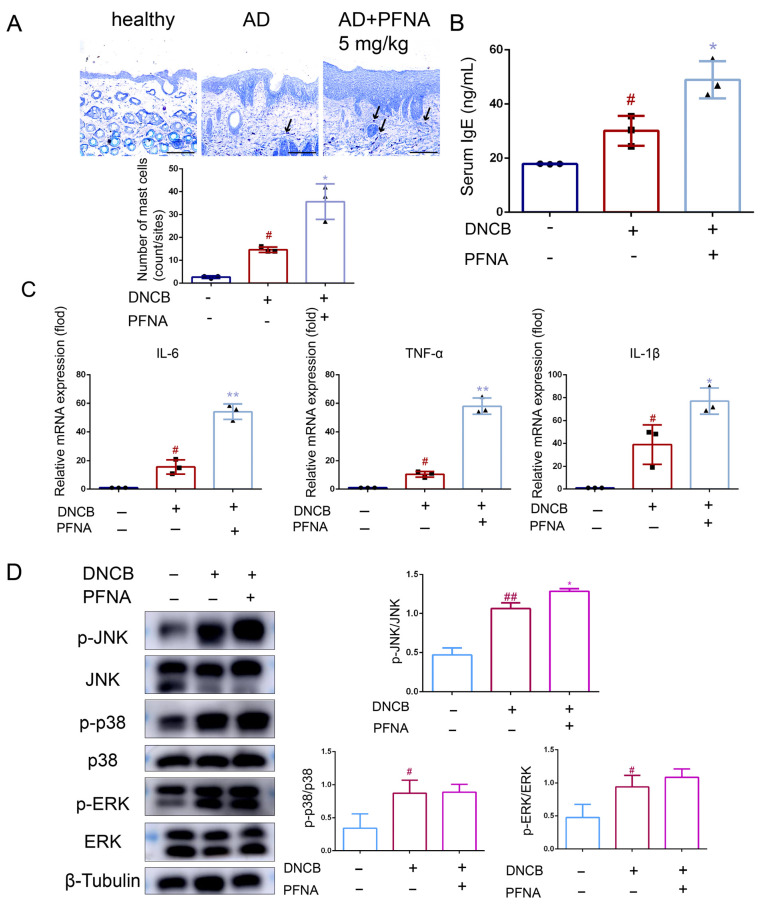
Effect of PFNA on DNCB-induced AD. (**A**) Representative histology (toluidine blue [TB] stain, magnification ×20, scale bar: 100 µm) and MC number (n = 3). Numbers of MCs within the skin were quantified in 300 × 300 μm. (**B**) Total serum IgE level (n = 3). (**C**) Enhancement of DNCB-induced pro-inflammatory mediator production in skin tissues by PFNA in an in vivo setting. RT-qPCR was used to determine the levels of IL-6, TNF-α, and IL-1β (n = 3). (**D**) Effect of PFNA on MAPK phosphorylation (n = 3). p-p38, p-JNK, and p-ERK were detected by Western blot. β-Tubulin was used as an internal control. Data in the plots are shown as the mean ± SD (n = 3). ^#^ *p* < 0.05 and ^##^ *p* < 0.01 vs. the healthy group, * *p* < 0.05 and ** *p* < 0.01 vs. the AD group.

Mast cell (MC) infiltration represents a hallmark feature of atopic dermatitis (AD). MCs identified via toluidine blue staining were localized in the dermis, and the effect of PFNA was evaluated (Figure 3A). Few MCs were observed in the healthy group, whereas a significant increase in MC numbers was noted in the AD group. Compared with the model group, the AD + PFNA group exhibited enhanced MC infiltration. The IgE level in the serum was upregulated for the AD mice, and such upregulation of IgE was more significant in the AD + PFNA group compared with the healthy group (Figure 3B). The pro-inflammatory effect of PFNA was further examined by measuring the expression levels of TNF-α, IL-6, and IL-1β in the skin. Compared with the healthy group, the mRNA levels of TNF-α, IL-6, and IL-1β in the AD group were significantly upregulated, as shown by RT-qPCR analysis, with those in the AD + PFNA group being more pronounced (Figure 3C). The MAPK family proteins (JNK, ERK1/2, and p38MAPK) involved in anti-inflammatory responses also play an important role in inflammatory diseases [16]. As shown in Figure 3D, the AD mice showed significantly upregulated levels of p-JNK, p-ERK1/2, and p-p38 MAPK, but no change in the total JNK1/2, ERK1/2, and p38 MAPK. The AD + PFNA group showed a significantly upregulated p-JNK/JNK level. In general, this result demonstrates that PFNA exhibited pro-inflammatory activity through the MAPK signaling pathway. We investigated the effect of PFNA on the activation of the NF-κB signaling pathway (Figure S1A). Western blot results showed that the expression of p-IκBα and p-p65 was significantly increased in the AD mice, but the PFNA group did not have a significant effect on the expression of p-IκBα and p-p65 in the skin. Similarly, we investigated the effect of PFNA on apoptosis (Figure S1B). Western blot results showed that the PFNA group significantly upregulated caspase-3 expression in the skin.

### 3.5. PFNA Promotes LPS-Activated RAW264.7 Macrophages’ Inflammatory Response

**Figure 4:**
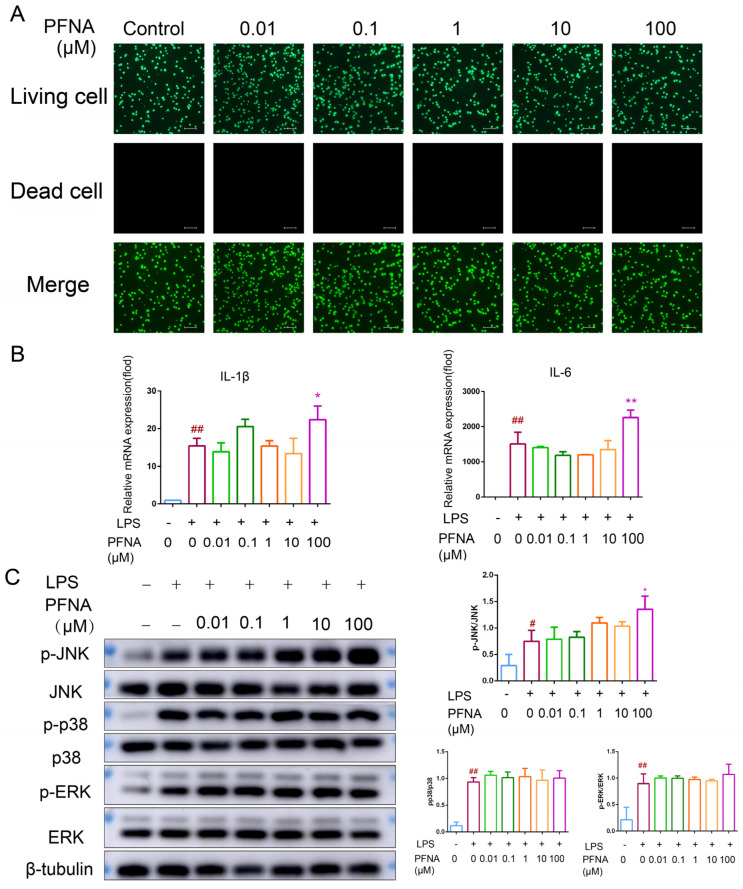
Pro-inflammatory effects of PFNA on LPS-stimulated RAW264.7 macrophages. (**A**) PFNA cytotoxicity in RAW264.7 macrophages. These cells were treated with indicated PFNA concentrations for 72 h, and cell viability was evaluated using calcein-AM/PI double fluorescent staining. (**B**) Effects of PFNA on the pro-inflammatory factor production by LPS-stimulated RAW264.7 macrophages. The cells were exposed to the indicated concentrations of PFNA for 72 h and then exposed to LPS (1 μg/mL) stimulation for 0.5 h. The mRNA levels of the pro-inflammatory factors IL-1β and IL-6 in the cells were measured by RT-qPCR. (**C**) Effects of PFNA on MAPK phosphorylation in LPS-stimulated RAW264.7 macrophages. RAW264.7 macrophages were exposed to the indicated concentrations of PFNA for 72 h, followed by exposure to 1 μg/mL LPS for 3 h. The levels of phosphorylated and total proteins for the indicated markers were determined by Western blot. β-Tubulin was used as an internal control. Data shown in each plot are the mean ± SD of three independent experiments. ^#^ *p* < 0.05 and ^##^ *p* < 0.01 vs. non-LPS and non-PFNA-treated, * *p* < 0.05 and ** *p* < 0.01 vs. LPS and non-PFNA-treated.

The pro-inflammatory effect of PFNA in vivo was further investigated by exploring the ability of PFNA to promote the inflammatory response of RAW 264.7 induced by LPS. An analysis of the cytotoxicity of RAW 264.7 by live–dead cell staining revealed no difference in cell viability among the different groups (Figure 4A). The pro-inflammatory effect of PFNA on LPS induction was subsequently evaluated by examining the changes in the mRNA levels of IL-6 and IL-1β in LPS-induced RAW264.7 cells in the absence and presence of PFNA. The levels of both IL-6 and IL-1β were markedly elevated in the LPS-stimulated group compared to controls, and PFNA pretreatment (100 μM) further augmented these cytokines relative to LPS stimulation alone (Figure 4B). In addition, Western blotting results showed that p-JNK/JNK, p-p38/p38, and p-ERK/ERK were significantly upregulated in the LPS-induced group, and the p-JNK/JNK ratio was significantly upregulated in the group treated with 100 μM PFNA compared with the group treated with just LPS (Figure 4C). These results indicate that high-dose PFNA can promote an inflammatory response.

### 3.6. PFNA Promotes Inflammatory Response in TNF-α-Activated bEnd.3 Cells

**Figure 5:**
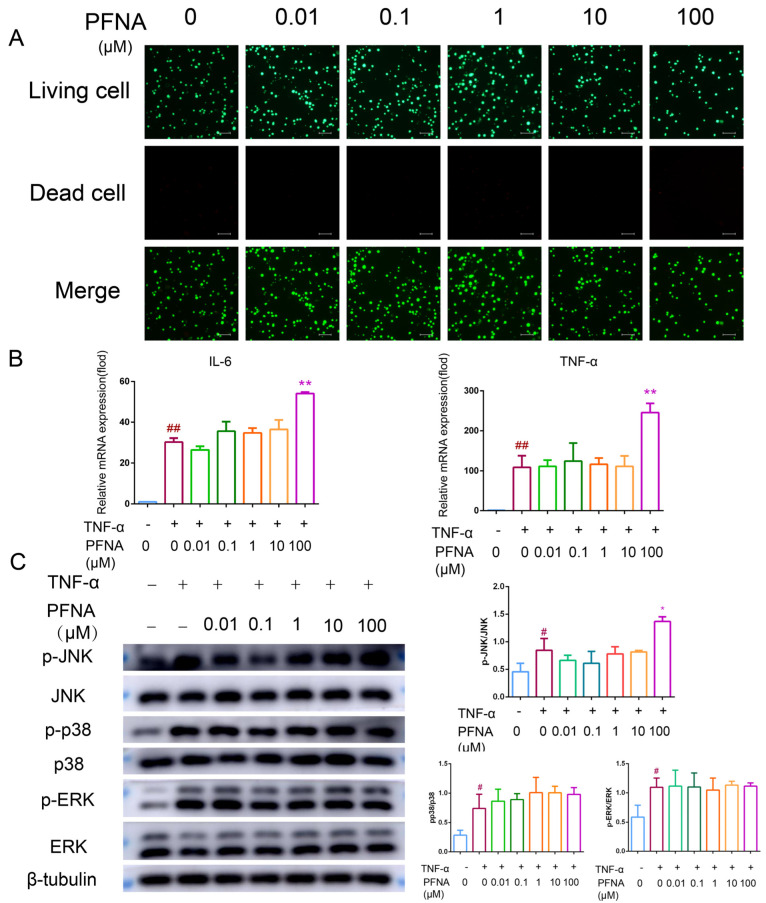
Pro-inflammatory effect of PFNA on TNF-α-stimulated bEnd.3 cells. (**A**) PFNA cytotoxicity in bEnd.3 cells visualized by calcein-AM/PI dual fluorescent staining. The bEnd.3 cells were exposed to the indicated concentrations of PFNA for 72 h before being stained with calcein-AM/PI for the observation of cell activity. (**B**) PFNA’s impact on pro-inflammatory factor generation in TNF-α-stimulated bEnd.3 cells. The bEnd.3 cells were exposed to the indicated concentrations of PFNA for 72 h, followed by exposure to TNF-α (20 ng/mL) stimulation for 0.5 h, and the mRNA levels of the pro-inflammatory factors IL-6 and TNF-α in the cells were measured by RT-qPCR. (**C**) Effect of PFNA on MAPK phosphorylation in TNF-α-stimulated bEnd.3 cells. The bEnd.3 cells were exposed to the indicated concentrations of PFNA for 72 h, followed by exposure to TNF-α (20 ng/mL) stimulation for 0.5 h. The levels of total and phosphorylated protein for the indicated markers were determined by Western blot. β-Tubulin was used as an internal control. Data shown in each plot are the mean ± SD of three independent experiments. ^#^ *p* < 0.05 and ^##^ *p* < 0.01 vs. non-TNF-α and non-PFNA-treated, * *p* < 0.05 and ** *p* < 0.01 vs. TNF-α and non-PFNA-treated.

The pro-inflammatory effect of PFNA was further examined using bEnd.3 cells. The cytotoxicity of bEnd.3 was first determined using live–dead cell staining, and no differences in the viability of the cells were detected among the different groups (Figure 5A). The RT-qPCR assay revealed a significant upregulation in the mRNA levels of IL-1β and IL-6 in the TNF-α-stimulated bEnd.3 cells relative to the control cells (Figure 5B), while the upregulation of TNF-α-induced IL-1β and IL-6 mRNAs was significant when the concentration of PFNA was 100 μM. Similarly, Western blot results showed that p-JNK/JNK, p-p38/p38, and p-ERK/ERK were significantly upregulated in the TNF-α-induced group, and the p-JNK/JNK ratio was significantly upregulated in the group treated with 100 μM PFNA (Figure 5C). These results indicate that high-dose PFNA can promote TNF-α-induced inflammatory responses in bEnd.3 cells.

### 3.7. PFNA Promotes Inflammatory Response in IFN-γ/TNF-α-Stimulated HaCaT Cells

The activation of keratinocytes plays an important role in the development and progression of AD [21,22]. We analyzed the levels of TNF-α and IL-1β mRNA in IFN-γ/TNF-α-induced HaCaT cells by RT-qPCR (Figure S2), and the mRNA levels of TNF-α and IL-1β were elevated in the AD cell model constructed from IFN-γ/TNF-α-induced HaCaT cells. The levels of the above inflammatory factors were further elevated after treatment of the AD cell model with PFNA (100 μM).

## 4. Discussion

This study investigated whether PFNA exacerbates AD symptoms using DNCB-induced AD-like lesions in BALB/c mice, LPS-induced inflammation in RAW264.7 macrophages, and TNF-α-stimulated inflammation in bEnd.3 cells. PFNA administration was found to exacerbate AD-like lesions and promote the expression of factors associated with inflammation in the dorsal skin of mice. In addition, PFNA also upregulated the production of pro-inflammatory factors and the expression of MAPK in the inflammatory response induced by LPS and TNF-α in the RAW264.7 and bEnd.3 cells, respectively. These findings provide support that PFNA can exacerbate the symptoms of atopic dermatitis.

MCs serve as critical immune cells, playing pivotal roles in the inflammatory dermatopathology of AD. AD is a chronic inflammatory skin disease characterized by the overproduction of IgE [23]. Some studies have indicated that IgE is a critical factor in the pathogenesis of AD, as well as a prognostic indicator of AD [24,25]. During allergic reactions, IgE increases and binds to high-affinity mast cells. The IgE-activated mobilization of mast cells releases mediators, including histamine, prostaglandins, and leukotrienes. In addition, mast cell overproduction leads to the infiltration of inflammatory epidermal cells, macrophages, and eosinophils [26,27], and MCs upregulate innate immune cells via cytokine release [28]. This study revealed that PFNA significantly upregulated MC degranulation and IgE levels, exacerbating AD-like symptoms. However, whether PFNA promotes innate immune inflammation remains to be explored. As shown by these results, it is suggested that there may be a health risk posed by PFNA regarding the symptoms of AD.

Previous studies have shown that the MAPK pathway is an important signal transfer pathway for activated inflammation [29]. MAPK is a serine/threonine kinase capable of sending extracellular signals to the nucleus and is activated in response to a variety of extracellular stimuli through phosphorylation on threonine and tyrosine residues [30], leading to an inflammatory response via the synthesis of mediators carried by its signaling [31]. The MAPK family comprises extracellular signal-regulated kinases (ERKs) along with the following two stress-responsive subgroups: c-Jun N-terminal kinase (JNK) and p38, both categorized as stress-activated protein kinase (SAPK) systems [32]. Several studies have shown the importance of MAPK in inducing inflammation [31,33,34,35]. Our results suggest that PFNA can promote the DCNB-, LPS-, and TNF-α-induced phosphorylation of JNK. These data suggest that modulation of the MAPK signaling pathway may represent a critical mechanistic component contributing to PFNA-mediated pro-inflammatory responses. NF-κB is also an important pathway in inflammation [36]. Exploring whether PFNA relies on the activation of this pathway to stimulate inflammation, the experimental results found that PFNA did not up-regulate NF-κB signaling, which is consistent with previous studies [37]. This may indicate that PFNA has a weak or no effect on the NF-κB pathway, and that the promotion of inflammatory responses mainly depends on the phosphorylation of JNK.

Apoptosis is involved in DNCB-induced skin damage [37], and the caspase pathway is the main pathway of apoptosis. In this experimental study, we found that PFNA promotes the protein expression of caspase-3 in AD mice, which suggests that PFNA can promote apoptosis to promote DNCB-induced atopic dermatitis.

In this study, we did not calculate the serum concentration of PFNA in the serum of mice, and the reference oral concentration was calculated according to Fang et al. [8] and Wang et al. [38]. According to the Fang [39] method, the serum concentration should be 10–200 times the human serum concentration, and this experiment was not tested according to the basic concentration in the human body. In order to test whether it has a pro-inflammatory effect on the deterioration of atopic dermatitis effects, the realistic application of PFNA needs additional experiments.

In the current study, it remains unclear how PFNA activates the MAPK pathway or whether PFNA directly binds to specific receptors on the membranes of RAW264.7 or bEnd.3 cells. Based on findings from the literature, PFNA may interact with the protein backbone via hydrogen bonds, enabling it to bind broadly to diverse proteins and significantly enhance their intracellular uptake [40]. For instance, PFBS (perfluorobutanesulfonic acid) has been shown to transmit signals through cell surface receptors, subsequently activating the MAPK cascade [41]. Further studies are required to investigate how PFNA enters these cells.

Short-term or long-term exposure to environmental pollutants can exacerbate atopic dermatitis symptoms [42]. In recent years, the immunotoxicity of PFNA has been significantly clarified. PFNA administered to BALB/c mice orally by gavage for 14 days [8] or as a single injection dose to C57BL/6 mice can cause significant lymphoid organ atrophy and alterations in the cellular structure of the spleen and thymus, as well as in the proportion of leukocyte populations, which triggers immunotoxicity in the organism [43]. In addition, PFNA can also increase the expression of inflammation-related factors [44], consistent with our earlier results showing that spleen atrophy and the upregulation of pro-inflammatory factors can occur in the PFNA-administered group. These previous results, therefore, provide support for the exacerbating effect of PFNA in atopic dermatitis mice.

In conclusion, our findings suggest the ability of PFNA to exacerbate DCNB-induced atopic dermatitis symptoms in BALB/c mice. PFNA damaged the spleen and upregulated the expressions of IL-6, IL-1β, and TNF-α inflammatory factors by activating the MAPK pathway. PFNA also exacerbated LPS-induced inflammation in RAW264.7 macrophages and TNF-α-induced bEnd.3 cells via the activation of the MAPK pathway, and could also increase skin cell apoptosis. Similarly, the present study found that PFNA promoted IFN-γ/TNF-α-induced inflammatory responses in HaCat cells. Collectively, PFNA may be an influential factor that could potentially exacerbate atopic dermatitis and worsen inflammation. Thus, further research is needed to understand the immunomodulatory mechanisms of PFNA and determine the potential health risks associated with exposure to this environmental contaminant.

## Figures and Tables

**Table 1:** The primer sequences for quantitative real-time PCR (*RT*-*qPCR*).

Gene	Species	Primer
GAPDH	Mouse	F: 5’-AGAAGGTGGTGAAGCAGGCATC-3’ R: 5’-CGAAGGTGGAAGAGTGGGAGTTG-3’
IL-1β	Mouse	F: 5’-TGTGTAATGAAAGACGGCACACC-3’ R: 5’-GTATTGCTTGGGATCCACACTCTC-3’
TNF-α	Mouse	F: 5’-AAGTTCCCAAATGGCCTCCCTCTC-3’ R: 5’-TCCTCCACTTGGTGGTTTGCTAC-3’
IL-6	Mouse	F: 5’-TCCTCTCTGCAAGAGACTTCCATC-3’ R: 5’-TGGTTGTCACCAGCATCAGTCC-3’

**Table 2:** The list of primary antibodies for western blot.

Antibody	Host	Dilution	Source	Cat#
JNK	Rabbit	1:1000	Cell Signaling Technology	#9252
p-JNK	Mouse	1:2000	Cell Signaling Technology	#9255
ERK	Rabbit	1:1000	Cell Signaling Technology	#4695
p-ERK	Rabbit	1:1000	Cell Signaling Technology	#4376
p-38	Rabbit	1:1000	Cell Signaling Technology	#8690
p-p38	Rabbit	1:2000	Proteintech	28796-1-AP
IκB-α	Rabbit	1:1000	Cell Signaling Technology	#9242
p-IκB-α	Mouse	1:1000	Cell Signaling Technology	#9246
p-65	Mouse	1:1000	Cell Signaling Technology	#6956
p-p65	Rabbit	1:1000	Cell Signaling Technology	#3033
Caspase-3	Rabbit	1:1000	Proteintech	25128-1-AP
β-Tubulin	Mouse	1:2000	Abmart	M30109
anti-mouse IgG		1:10,000	Jackson	115-035-003
anti-rabbit IgG		1:10,000	Jackson	111-035-003

## Data Availability

Dataset is available upon request from the authors.

## Supplementary Materials

The following supporting information can be downloaded at: https://www.mdpi.com/article/10.3390/toxics13070585/s1, Figure S1. Effects of PFNA on NF-κB signalling pathway and apoptotic proteins in AD mice. Figure S2. The effect of PFNA on the mRNA expression levels of cytokines in IFN-γ/TNF-α-stimulated HaCaT cells.
