# Supplementary material for: Perfluorononanoic Acid (PFNA) Exacerbates Atopic Dermatitis by Inducing Inflammation in Mice
Source: Toxics. 2025 Jul 13;13(7):585. doi: 10.3390/toxics13070585 (PMC12298299; doi:10.3390/toxics13070585)
Supplement: Supplementary file 1 [file toxics-13-00585-s001.zip › toxics-3661910-supplementary.pdf]

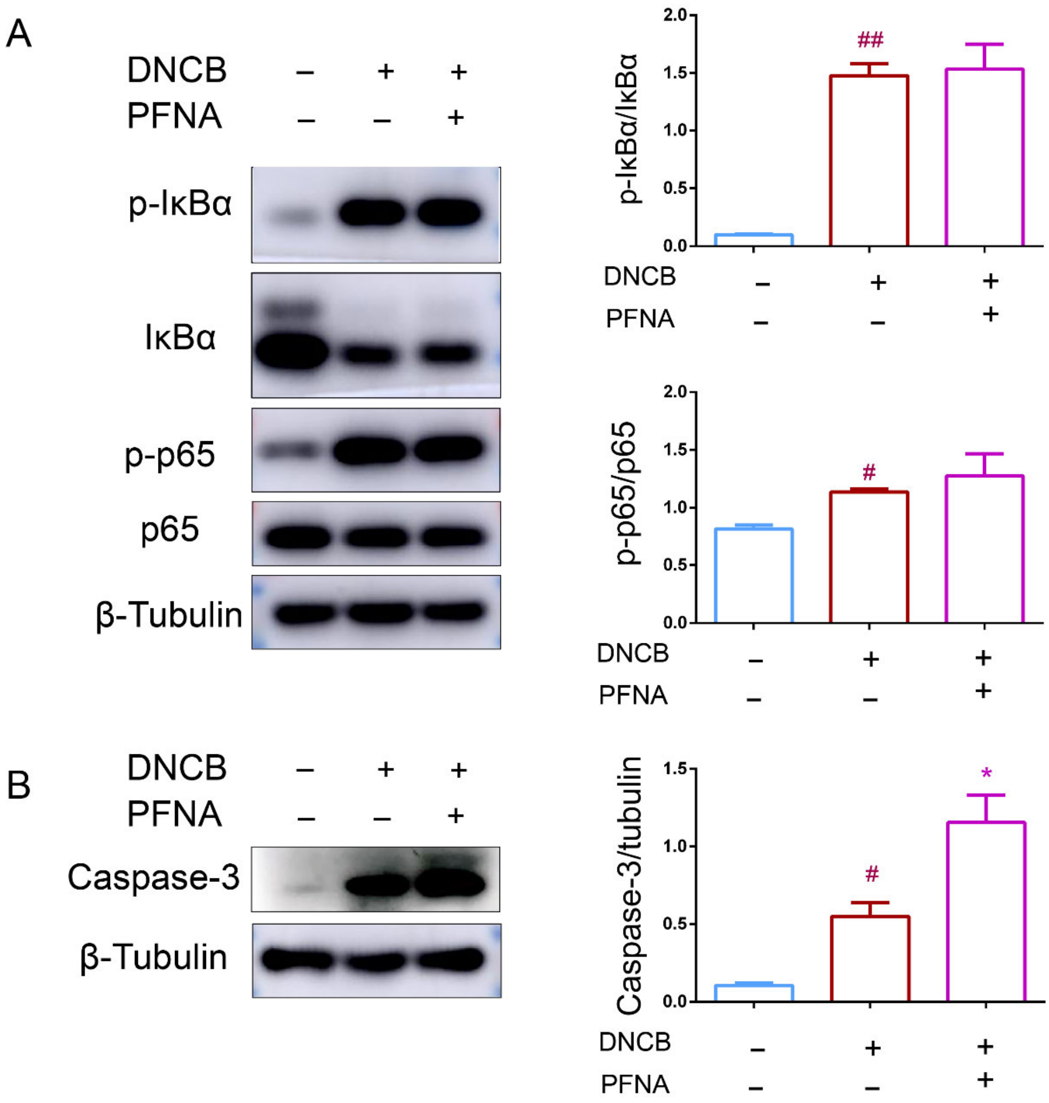

FigureS1.Effects of PFNA on NF-κB signalling pathway and apoptotic proteins in AD mice. (A)Effect of PFNA on NF-κB phosphorylation. (n = 3) p-p38, p-IκBα, IκBα, p65 and p-p65 were detected by western blot. (B) Caspase-3 were detected by western blot. β-Tubulin was used as an internal control. Data in the plots are shown as the mean ± SD (n = 3). $^{\#}$ *P*<0.05, $^{\#\#}$ *P*<0.01 vs. the healthy group, * *P*<0.05 vs. the AD group.

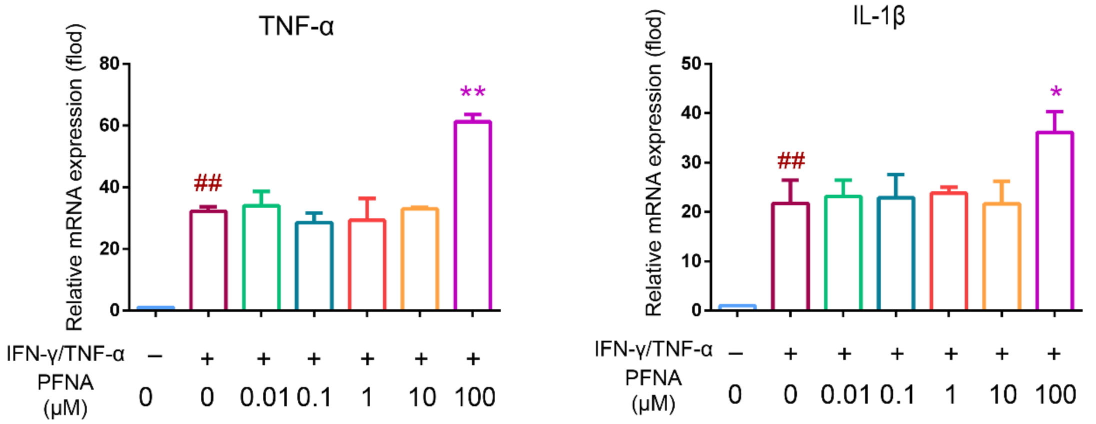

FigureS2.The effect of PFNA on the mRNA expression levels of cytokines in IFN-γ/TNF-α-stimulated HaCaT cells. HaCaT cells were pretreated with different concentrations of PFNA (0.01,0.1,1,10 and 100 μM) for 72 h, and then the cells were treated with IFN-γ/TNF-α (10 ng/mL) for 1 h. Total RNA was isolated, and the mRNA levels of the pro-inflammatory factors TNF-α and IL-1β in the cells were measured by qPCR. Data shown in each plot are the mean ± SD of three independent experiments. $^{\#\#}$ $P < 0.01$ vs. non-IFN-γ/TNF-α, and non-PFNA-treated, * $P < 0.05$, ** $P < 0.01$ vs. IFN-γ/TNF-α and non-PFNA-treated.
